# Upconversion‐Luminescent Fiber Microchannel Sensors for Temperature Monitoring at High Spatial Resolution in the Brains of Freely Moving Animals

**DOI:** 10.1002/advs.202303527

**Published:** 2023-09-15

**Authors:** Bingqian Zhou, Kuikui Fan, Jiazhen Zhai, Cheng Jin, Lingjie Kong

**Affiliations:** ^1^ State Key Laboratory of Precision Measurement Technology and Instruments Department of Precision Instrument Tsinghua University Beijing 100084 China; ^2^ IDG/McGovern Institute for Brain Research Tsinghua University Beijing 100084 China

**Keywords:** distributed temperature probes, fiber microchannel sensors, high spatial resolution, in vivo, upconversion nanoparticles

## Abstract

Brain temperature is a critical factor affecting neural activity and function, whose fluctuations may result in acute life‐threatening health complications and chronic neuropathology. To monitor brain temperature, luminescent nanothermometry (LN) based on upconversion nanoparticles (UCNPs) with low autofluorescence has received extensive attention for its advantages in high temperature sensitivity and high response speed. However, most of current the LNs are based on optical imaging, which fails in temperature monitoring in deep brain regions at high spatial resolution. Here, the fiber microchannel sensor (FMS) loaded with UCNPs (UCNP‐FMS) is presented for temperature monitoring at high spatial resolution in the deep brains of freely moving animals. The UCNP‐FMS is fabricated by incorporating UCNPs in microchannels of optical fibers, whose diameter is ∼50 µm processed by femtosecond laser micromachining for spatially resolved sensing. The UCNPs provide thermal‐sensitive upconversion emissions at dual wavelengths for ratiometric temperature sensing, ensuring a detection accuracy of ± 0.3 °C at 37 °C. Superior performances of UCNP‐FMS are demonstrated by real‐time temperature monitoring in different brain regions of freely moving animals under various conditions such as taking food, undergoing anesthesia/wakefulness, and suffering external temperature changes. Moreover, this study shows the capability of UCNP‐FMS in distributed temperature sensing in mammalian brains in vivo.

## Graphical abstract

A fiber microchannel sensor loaded with upconversion nanoparticles (UCNPs) is applied for temperature monitoring at high spatial resolutions with a high accuracy of ∼0.3 °C in the deep brains of freely moving animals. Combined with two UCNPs, the dual microchannel probes can simultaneously detect brain temperatures at different depths in vivo.

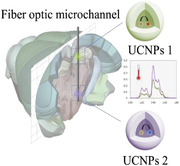

## 1 Introduction

Brain temperature, a metabolism‐related parameter and a factor affecting neural activity and function is regarded as a tightly regulated and highly stable homeostatic parameter. Besides, recent reports find that brain temperature distribution and thermal dynamics are relevant parameters for understanding brain physiology^[^ ^1^ ^]^ and neuropathology, potentially as diagnostic indicators.^[^ ^2^ ^]^ For example, Petersen et al. reported that brain temperature showed wide fluctuations (∼3 °C) across natural behaviors.^[^ ^3^ ^]^ And local brain temperature is regarded as an important indicator of neural activity. Fekete et al. reported that heating and cooling of local brain regions could temporarily activate and silence neural activity in a transgene‐free manner.^[^ ^4^ ^]^

Despite increasing attentions from clinicians, the distribution, fluctuations, and changes of brain temperatures in response to external stimuli are still largely unknown and difficult to measure with conventional temperature detection methods. Physiological studies of brain temperature changes often rely on the implantation of thermocouples or thermistors, which exhibit high sensitivity and accuracy, but these approaches are susceptible to electromagnetic interference.^[^ ^5^ ^]^ Optical temperature sensors, such as infrared spectroscopy and optical fibers employing Bragg gratings or doping rare earth ions, are immune to electromagnetic noise, which can be employed to detect tissue temperature.^[^ ^6^, ^7^ ^]^ But these approaches are limited to point measurements at low spatial resolutions, failing in measuring brain temperature distribution.^[^ ^8^ ^]^ Magnetic resonance spectroscopy (MRS) can be used to detect small temperature variations (<1 °C) in the brains, however, the high cost of MRS instruments and measurement artifacts become crucial barriers for practical measurements.^[^ ^9^ ^]^ Therefore, it is still urgent to design temperature sensors of low‐cost, while guaranteeing high thermal, spatial, and temporal resolutions.

Recently, a series of organic and inorganic nanoscale thermometers have been designed for temperature sensing based on fluorescence imaging, to achieve high thermal, spatial, and temporal resolutions.^[^ ^10^ ^]^ Compared with thermometers of organic fluorescent dyes, inorganic luminescent nanothermometry has good photostability and avoids the problem of photobleaching.^[^ ^11^ ^]^ In which, lanthanum (Ln^3+^) ‐doped upconversion nanoparticles (UCNPs) are promising with excellent temperature response, high brightness, excellent photochemical stability, and long luminescence lifetimes.^[^ ^12^ ^]^ Besides, owing to large anti‐Stokes shifts, signals from UCNPs are immune to auto‐fluorescence from biological systems.^[^ ^13^, ^14^, ^15^ ^]^ Thus, temperature imaging based on various UCNPs has been performed to monitor temperature changes in living cells, tissues, and animals.^[^ ^16^, ^17^, ^18^, ^19^ ^]^ For example, Di et al. monitored the thermodynamics of lysosomes and mitochondria with modified UCNPs, showing the capability in detecting temperature dynamics at the organelle level.^[^ ^20^ ^]^ Unfortunately, the thermal sensitive signals of these upconversion luminescent nanothermometers are mostly located in the visible region, which limits the detection depth in optical imaging due to high absorption and scattering in biological tissues. To increase the imaging depth, some new temperature‐sensitive nanomaterials have been developed, which convert luminescence from visible to near‐infrared (NIR), such as time‐resolved nanothermometry^[^ ^21^ ^]^ and Ag_2_S nanothermometers.^[^ ^22^ ^]^ Nevertheless, temperature detection of deep brain regions based on nanothermometry in freely moving animals has not been achieved yet, due to the confinement of imaging.^[^ ^10^ ^]^ Optical fiber‐based spectral detection combined with sol–gel technology provides an alternative way to detect the temperature in vivo,^[^ ^19^, ^23^ ^]^ especially in deep brain regions of freely moving creatures, but the distributed thermal sensing with high spatial resolutions is still challenging.

**Figure 1:**
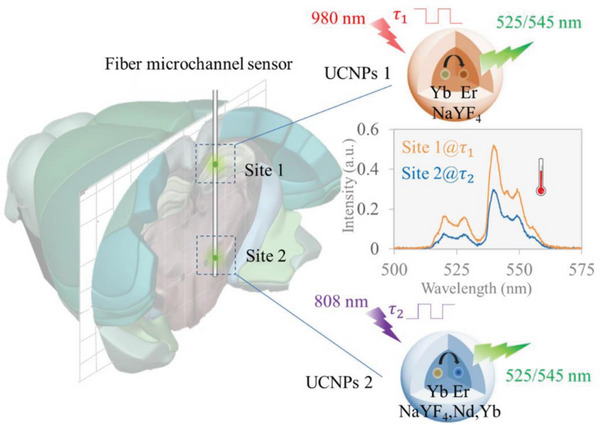
Schematic diagram of temperature sensing in vivo with the UCNP‐FMS. UCNP‐FMS is implanted in the mouse brains for measuring thermal dynamics. The two kinds of UCNPs are loaded in the microchannels of optical fibers. UCNPs 1 denotes NaYF_4_:Yb,Er@NaYF_4_ nanoparticles, emitting 525/545 nm fluorescence under 980 nm laser excitation for ratiometric temperature detection at Site 1. UCNPs 2 denotes NaYF_4_:Yb,Er@NaYF_4_:Nd,Yb nanoparticles, emitting 525/545 nm fluorescence under 808 nm laser excitation for ratiometric temperature detection at Site 2. Through time division multiplexing, dual‐site temperature detection is achieved. τ_1_ and τ_2_ denote the time modulations of 980 and 808 nm laser.

Here, we propose a fiber microchannel sensor (FMS) loaded with UCNPs (UCNP‐FMS) for temperature monitoring at high spatial resolutions in the brains of freely moving animals (**Figure** **1**). The UCNP‐FMS is fabricated by incorporating UCNPs in optical fiber microchannels, whose diameter is ∼50 µm by ultra‐high precision femtosecond laser fabrication. The laser micromachining not only ensures high spatial resolutions in distinguishing temperatures of different brain regions but also enables distributed temperature sensing with multiple microchannels filled with various UCNPs along the optical fibers. The UCNPs doped with Nd^3+^, Yb^3+^, and Er^3+^, generate thermal‐sensitive dual‐wavelength emissions under NIR excitation, enabling the ratiometric readout for self‐calibrated temperature sensing. Thermal characterizations of the NaYF_4_:Yb,Er@NaYF_4_ UCNP‐FMS sensor show a reversible and stable response to the large range of temperature changes (35–80 °C), a rapid responsivity (∼10 s), and a low detection limit (± 0.3 °C @ 37 °C), suitable for detecting subtle changes of brain temperature. We perform a series of in vivo experiments to demonstrate the performance of UCNP‐FMS in monitoring temperatures of different brain regions at high spatial resolutions in freely‐moving mice, under different conditions such as taking food, undergoing anesthesia/wakefulness, and suffering external temperature changes. Moreover, we demonstrate UCNP‐FMS with two microchannels loading two thermal‐sensitive UCNPs separately, i.e., NaYF_4_:Yb,Er@NaYF_4_ and NaYF_4_:Yb,Er@NaYF_4_:Nd,Yb, for distributed temperature detection at different depths in the brains of freely moving mice. These suggest that UCNP‐FMS is promising for applications in brain physiology, pathology, and pharmacology research.

## 2 Results and Discussion

### 2.1 Characterization of Temperature‐Sensitive UCNPs

**Figure 2:**
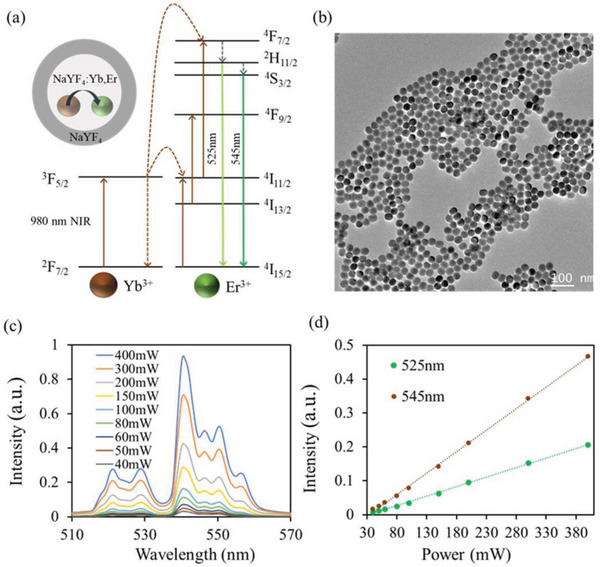
Characterization of temperature‐sensitive NaYF_4_:Yb,Er@NaYF_4_ UCNPs. a) Energy level diagram of the upconversion process of the NaYF_4_:Yb,Er@NaYF_4_ UCNPs. The UCNPs consist of an active core (NaYF_4_:Yb,Er) and an inert coating shell (NaYF_4_). b) TEM image of the NaYF_4_:Yb,Er@NaYF_4_ nanoparticles. Scar bar: 100 nm. c) Emission spectra of the NaYF_4_:Yb,Er@NaYF_4_ UCNPs under 980 nm excitation at different excitation powers. The concentrations of the UCNPs are set at 0.1% w v^−1^ dispersed in cyclohexane. d) Linear relationships between emission intensity (@ 525 and 545 nm) and 980 nm excitation laser power of the NaYF_4_:Yb,Er@NaYF_4_ UCNPs (*n* = 3). Data are presented as mean ± standard error of mean (SEM), *n* = 3 per group.

Thermal sensitive Ln^3+^‐doped UCNPs are synthesized and microinjected into the microchannels of FMS for temperature sensing. **Figure** **2**a shows the schematic diagram of the upconversion process of the NaYF_4_:Yb,Er@NaYF_4_ nanoparticles. The core of UCNPs (NaYF_4_:Yb,Er) provides visible emissions under 980 nm excitations via energy transfer from the Yb^3+^ ions to Er^3+^ ions, and the inert shell (NaYF_4_) protects the active dopant ions from nonradiative decay caused by surface defects and thus improves the upconversion emissions. The NaYF_4_:Yb,Er@NaYF_4_ UCNPs are synthesized via the solvothermal method and the obtained UCNPs are dispersed in cyclohexane.^[^ ^24^ ^]^ The transmission electron microscopy (TEM) image of the NaYF_4_:Yb,Er@NaYF_4_ UCNPs is shown in Figure 2b, which shows uniform hexagonal morphology with an average diameter of 30 nm. Distinguished from other fluorophores such as organic dyes and quantum dots, Ln^3+^‐doped UCNPs offer highly attractive advantages in narrow emission peaks and excellent photostability.^[^ ^25^ ^]^ Figure 2c shows the upconversion emission spectra of the NaYF_4_:Yb,Er@NaYF_4_ UCNPs, which exhibit two thermal sensitive emission bands centered at 525 and 545 nm under 980 nm excitation, corresponding to the ^2^H_11/2_→^4^I_15/2_ and ^4^S_3/2_→^4^I_15/2_ transitions of Er^3+^, respectively. When the excitation power is increased from 40 to 400 mW, a linear increase in the peak emission intensities (@ 525 and 545 nm) of UCNPs is observed, as shown in Figure 2d.

### 2.2 Fabrication of UCNP‐FMS Probe

**Figure 3:**
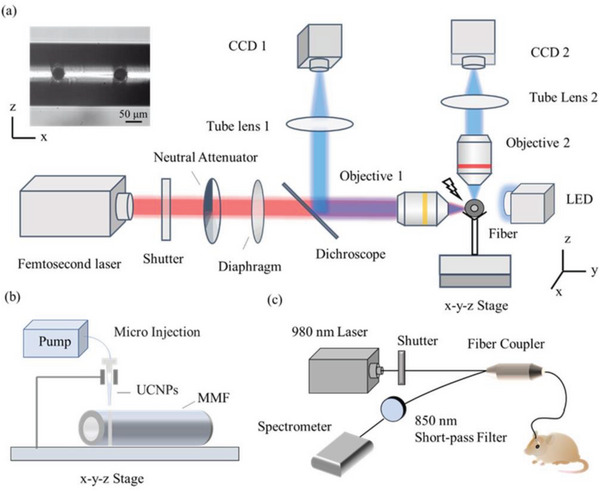
Fabrication of the UCNP‐FMS probe and the temperature sensing system. a) Optical setup for fabricating fiber microchannels by femtosecond laser micromachining. Inset: a photo of fiber microchannels in the *x‐z* plane. The focal lengths of tube lens 1 and tube lens 2 are 100 mm. Objective 1: 10 × , NA 0.25, working distance 7.4 mm. Objective 2: 4 × , NA 0.1, working distance 27.2 mm. LED: Light emitting diode, 473 nm, used as an illumination light source. CCD 1, 2: Charge‐coupled device cameras, imaging the processed fiber in the *x‐y* plane and *x‐z* plane, respectively. b) Microinjection of UCNPs into a microchannel. MMF: multimode fiber. c) Optical setup for temperature sensing based on UCNP‐FMS.

The fabrication of the UCNP‐FMS probe is mainly divided into two steps. First, the fiber microchannel is fabricated by femtosecond laser micromachining on the silica multi‐mode fiber (MMF), which ensures high precision, high quality, and high spatial‐resolution.^[^ ^26^, ^27^, ^28^ ^]^ By translating the silica optical fiber with respect to the focal point of the focused femtosecond pulse, femtosecond laser micromachining can be used to fabricate 3D structures,^[^ ^29^ ^]^ and it does not require clean room facilities making it a fast, simple and cost‐effective procedure.^[^ ^30^ ^]^ Here, we fabricate microchannels with ∼50 µm diameters in the front end of the MMF (**Figure** **3**a). A femtosecond laser (Coherent Legend Elite) emitting at 800 nm is tightly focused on the targeted positions of MMF (core/cladding 200/220 µm) using a 10 × objective with a numerical aperture (NA) of 0.25. The shutter controls the processing time, the neutral attenuator is used to control the power of the laser, and the diaphragm is used to improve the quality of the focused light spot. The MMF is fixed on a 3D mobile platform, so that we can write desired structures by translating it with respect to the laser beam at a writing speed of 10 µm s^−1^. Two cameras are employed for observing the machining position at two sections of the processed fiber to ensure that the microchannel is located at the fiber axis and runs through the entire section. The detailed optimized processes of femtosecond laser micromachining can be found in the supporting information (Figures S1 and S2, Supporting Information).

Then, with the aid of a stereoscope, we use a micro syringe to inject the UCNPs (2.5 mg ml^−1^, 40 nl) to the microchannels (Figure 3b). Since the UCNPs are dispersed in cyclohexane, after a period of evaporation, only the UCNPs remain in the microchannel. To prevent the leakage of UCNPs, the microchannel can be sealed with UV adhesive. The UCNP‐FMS is stored in dry air before use. As shown in Figure 3c, we develop a compact optical setup for temperature monitoring based on UCNP‐FMS. A 980 nm continuous wave laser is modulated by a shutter to reduce the thermal effect caused by constant luminescence and launched into UCNP‐FMS through a 50:50 fiber coupler. The upconversion emission is collected and back‐propagates to a spectrometer for spectral analysis after short‐pass filtering (cut‐off wavelength 850 nm). The UCNP‐FMS is implanted in mouse brains for real‐time temperature monitoring in vivo.

### 2.3 In vitro Temperature Sensing with UCNP‐FMS

**Figure 4:**
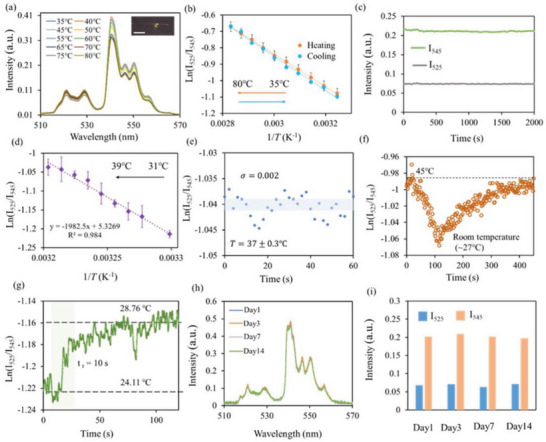
In vitro temperature sensing with UCNP‐FMS. a) Emission spectra of the UCNP‐FMS at various temperatures under 980 nm laser excitation (20 mW). Inset is the photo of UCNP‐FMS probe, and scale bar is 1 mm. b) Plot of ln(I_525_/I_545_) versus the inverse absolute temperature (1/*T*) during both heating and cooling processes (*n* = 3). c) Photostability of the FMS doped with NaYF_4_:Yb,Er@NaYF_4_ UCNPs. d) Calibration of the UCNP‐FMS in the temperature range of 31–39 °C (*n* = 3). e) Fluctuations of the sensor output over time at the constant temperature of 37 °C. f) Response curve of the UCNP‐FMS when external temperature changes between 45 °C and room temperature. g) Response curve of the UCNP‐FMS under laser heating. h,i) Long‐term stability of the UCNP‐FMS. The sensor is kept in an incubator at a constant temperature of 37°C, h) the emission spectra and i) intensities of two emission bands are recorded for 14 successive days. Data are presented as mean ± SEM, *n* = 3 per group for (b) and (d).

We apply UCNP‐FMS for temperature sensing in vitro. **Figure** **4**a shows the detection spectra of the UCNP‐FMS under 35–80 °C, which are highly dependent on the temperature attributed to the thermally coupled energy states of Er^3+^. The ^2^H_11/2_→^4^I_15/2_ and ^4^S_3/2_→^4^I_15/2_ transitions are in close proximity that leads to a thermal equilibrium governed by the Boltzmann factor,^[^ ^31^, ^32^ ^]^ described as

(1) $$I_{525}/I_{545}=C\;\exp\left(-\Delta E/kT\right)$$

where I_525_ and I_545_ are the emission intensities of the ^2^H_11/2_→^4^I_15/2_ and ^4^S_3/2_→^4^I_15/2_ transitions, respectively; C is a constant determined by the host material of UCNPs; ΔE is the energy gap between the ^2^H_11/2_ and the ^4^S_3/2_ states; k is the Boltzmann constant; *T* is the absolute temperature (in Kelvin scale). Figure 4b shows the linear plots of ln(I_525_/I_545_) versus the inverse of the absolute temperature (1/*T*) in the temperature range of 35–80 °C, during both the heating and cooling processes. It indicates reversible and overlapped trends with no hysteresis in response to temperature increasing and decreasing. We verify the long time photostability of the sensor by testing it for more than half an hour, and prove the ability for detecting temperature quantitatively over a long period of time (Figure 4c). The ratiometric measurement is intrinsically self‐calibrated and thus makes the sensor robust to environmental perturbations such as animal motions. We calibrate the sensor over the temperature range of 31–39 °C (covering the normal brain temperature^[^ ^1^ ^]^), which shows a linear trend, well fitted as ln (I_525_/I_545_) = −1982.5 × (1/*T*) + 5.3269, shown in Figure 4d. The sensitivity of the sensor is defined as

(2) $$S=\delta \left[\ln\left(I_{525}/I_{545}\right)\right]/\delta T=-\Delta E/k\cdot \left(1/T^{2}\right)$$

where S is the sensitivity; δ[ln (I_525_/I_545_)] is the variation of the logarithm of the fluorescence ratio; δ*T* is the variation of the absolute temperature; ΔE, k, and *T* are the same as Equation (1). According to the Equation (2), the fluctuation of the sensor readout at a constant temperature of 37 °C proves the detection limit is about ± 0.3 °C, estimated from the standard deviation of signal drifting (Figure 4e). As shown in Figure 4f, the response time of the sensor is ∼100 s when we remove it from the incubator (∼45°C) and let it cool at the room temperature (∼27 °C). After cooling to room temperature, the sensor output recovers to the baseline when put back to 45 °C. The long response time is mainly caused by heat exchange between the external environment and the probe. To validate that the probe has a rapid response time when the temperature changes in a small range, we focus the 1040 nm femtosecond laser (∼700 mW) at the microchannel of UCNP‐FMS, and find that the temperature rises ∼4.65 °C within 10 s (Figure 4g). To test the long‐term stability, the sensor is kept at a constant temperature of 37 °C, and the emission spectra are recorded for 14 successive days (Figure 4h), which suggests little changes in emission intensity of the sensor except for ∼0.05 drifts (Figure 4i). The in vivo stability of the UCNP‐FMS is tested by long‐term temperature recording in mouse brains for a week (Figure S3, Supporting Information).

### 2.4 In vivo Temperature Sensing with UCNP‐FMS

**Figure 5:**
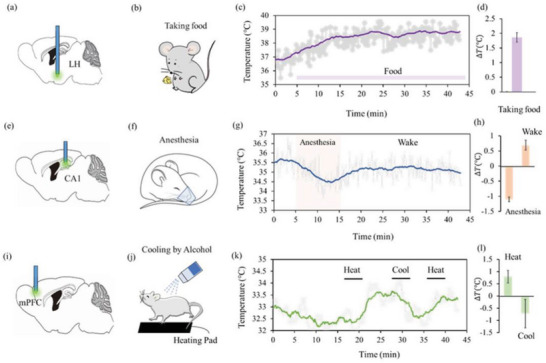
In vivo temperature sensing with UCNP‐FMS in the brains of freely moving mice. a) UCNP‐FMS implantation in lateral hypothalamus (LH) of mouse brains. b) Schematic of food intake. c) Temperature dynamics during a mouse is taking food. d) Temperature variation of LH in mouse brains during taking food (*n* = 3). e) UCNP‐FMS implantation in CA1 of mouse brain. f) Schematic of introducing anesthesia. g) Temperature dynamics of a mouse during the anesthesia and wakefulness cycle. h) Temperature variation of CA1 in mouse brains during anesthesia/wakefulness cycle (*n* = 3). i) UCNP‐FMS implantation in medial prefrontal cortex (mPFC) of mouse cerebral cortex. j) Schematic of cooling and heating the mouse body. k) Temperature dynamics during a mouse in cooling/heating environments. l) Temperature variation of mPFC in mouse brains during cooling and heating (*n* = 3). Emission spectra of the UCNP‐FMS are recorded under 980 nm laser excitation (20 mW). For (c), (g), and (k), the dark solid lines represent the smooth results (the smoothing window: 1000 s), and the light gray dotted lines represent raw data collected every 10 s. Data are presented as mean ± SEM, *n* = 3 mice per group for (b), (h), and (l).

Cerebral temperature reflects metabolic activity and can serve as an indicator of many diseases. It is known that the lateral hypothalamus (LH) is involved in regulating the autonomic nervous system, such as metabolism, body temperature, feeding, sleep, etc. Thus, we implant UCNP‐FMS in the LH of mouse brains (**Figure** **5**a), and monitor temperature changes when the mice are in the process of taking food (Figure 5b–d). We observe a slow increase of temperature, starting from ∼36.8 to ∼38.8 °C, i.e., ∼2 °C increase in LH, similar to the report of Bai et al.^[^ ^33^ ^]^ This verifies the ability of UCNP‐FMS in thermal monitoring in the deep brain regions of freely moving animals.

With the superior performance of UCNP‐FMS, we study the strong link between brain temperature and activity. As a proof of concept, we implant UCNP‐FMS in the CA1 of hippocampus in the mouse brain, and monitor brain temperature during anesthesia and wakefulness, as shown in Figure 5e. It is reported that isoflurane can cause a reduction in brain temperature,^[^ ^34^ ^]^ so we monitor CA1 temperature before, during, and after isoflurane administration (Figure 5f). During anesthesia, the brain temperature at CA1 goes down from 35.5 to 34.5 °C, with a slight decrease of ∼1 °C. After isoflurane administration, the mouse gradually recovers, and the brain temperature at CA1 increases ∼0.5 °C (Figure 5g,h). This shows that the brain temperature is related to brain activity, consistent with other reports.^[^ ^22^ ^]^

In addition, we investigate the effects of external cool and hot stimuli on brain temperature. We implant UCNP‐FMS in the medial prefrontal cortex (mPFC) of a mouse to monitor brain temperature dynamics during the cool and hot circulation (Figure 5i). To determine the dynamic changes of brain temperature caused by environmental changes, we first place the mouse in a heating pad (37 °C) for 5 min, then remove the heating pad and gently treat the mouse body (except for the head) with atomized ethanol for 5 min (Figure 5j). Again, we repeat the heat treatment for 5 min. We find that the brain temperature of mouse cortex gradually increases from 33 to 33.8 °C, then decreases to 32.6 °C, followed by increasing to 33.5 °C (Figure 5k). During the whole process, the brain temperature at cortex is highly correlated with external changes, with an average change of about ± 0.8 °C (Figure 5l).

In order to verify the accuracy of temperature detected by UCNP‐FMS in vivo, we implant a commercial thermocouple (YET‐620, YOWEXA) and the UCNP‐FMS into the cerebral cortexes of the same anesthetized mice. There is no significant difference in temperature detected between these two methods, which further demonstrates the robustness of our sensors (Figure S4, Supporting Information).

### 2.5 Distributed Temperature Sensing at High Spatial Resolution with UCNP‐FMS

**Figure 6:**
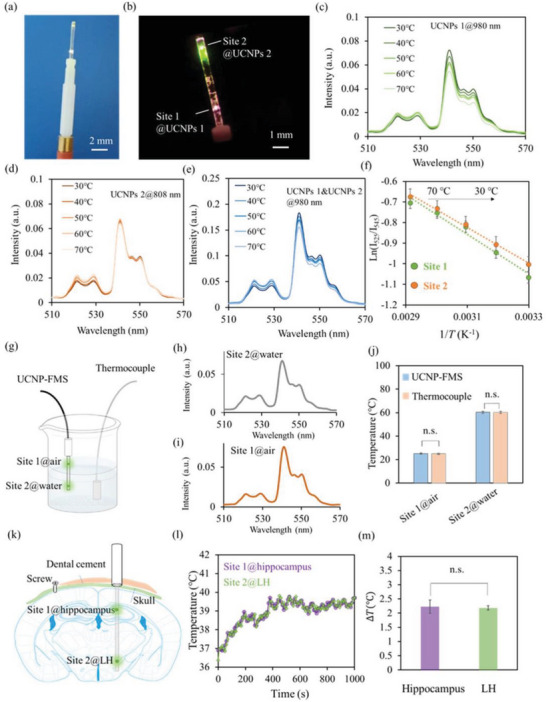
Distributed temperature sensing in vitro and in vivo with UCNP‐FMS based on NaYF_4_:Yb,Er@NaYF_4_ and NaYF_4_:Yb,Er@NaYF_4_:Nd,Yb. a) Photo of the UCNP‐FMS loading two UCNPs at seperate microchannels. b) Fluorescence of the UCNP‐FMS loading two UCNPs excited by 808 nm laser. UCNPs 1: NaYF_4_:Yb,Er@NaYF_4_, UCNPs 2: NaYF_4_:Yb,Er@NaYF_4_:Nd,Yb. c) Emission spectra of the UCNP‐FMS loading only UCNPs 1 at various temperatures under 980 nm laser excitation. d) Emission spectra of the UCNP‐FMS doped with two UCNPs at various temperatures under 808 nm laser excitation. e) Emission spectra of the UCNP‐FMS doped with two UCNPs at various temperatures under 980 nm laser excitation. f) Linear relationships of UCNPs at two sites between ln(I_525_/I_545_) and the inverse absolute temperature (1/*T*) in the range of 30–70 °C (*n* = 3). g) Schematic of in vitro temperature detection of the UCNP‐FMS. Site1@air denotes the upper microchannel is in air. Site2@water denotes the lower microchannel is in water. h) Spectrum of the UCNP‐FMS at Site2 under 808 nm laser excitaion. i) Demodulated spectrum of UCNP‐FMS at Site1 under 980 nm laser excaitaion. j) In vitro temperature sensing in two regions with UCNP‐FMS and thermocouple (*n* = 3). k) Schematic of the implantation of UCNP‐FMS in mouse brains. Site1@ hippocampus denotes the upper microchannel is at hippocampus. Site2@LH denotes the lower microchannel is at the LH. l) in vivo temperature sensing in two brain regions of freely‐moving mice with UCNP‐FMS. Temperature dynamics of hippocampus and LH during a mouse taking food are recorded. m) Temperature variations of hippocampus and LH in mouse brains during taking food (*n* = 3). The excitation power of 980 and 808 nm laser are 15 mW. Data are presented as mean ± SEM, *n* = 3 per group for (f) and (j), *n* = 3 mice per group for (m). P values: n.s. denotes p > 0.05 by one‐way analysis of variance (ANOVA) analysis of variance.

Thermal sensitive lanthanide (Ln^3+^)‐doped UCNPs have various excitation processes, many of which are suitable for temperature sensing. The characterization of the NaYF_4_:Yb,Er@NaYF_4_:Nd,Yb nanoparticles is shown in Figure S5 (Supporting Information), verifying the thermal sensing ability under 808 nm NIR excitation.^[^ ^35^, ^36^ ^]^ Therefore, we dope NaYF_4_:Yb,Er@NaYF_4_ UCNPs, and NaYF_4_:Yb,Er@NaYF_4_:Nd,Yb UCNPs into two microchannels separately along a FMS probe (**Figure** **6**a). This makes it possible to monitor temperatures at distributed positions with a single FMS probe under different NIR excitations, and the optical setup is shown in Figure S6 (Supporting Information). Under 808 nm laser excitation, only NaYF_4_:Yb,Er@NaYF_4_:Nd,Yb UCNPs emit fluorescence (Figure 6b), however, 980 nm laser excites both upconversion luminescence of the two UCNPs (Figure S7, Supporting Information), which needs spectral demodulation (Figure S8, Supporting Information) as follows. Briefly, the probe doped with the first kind of UCNPs (NaYF_4_:Yb,Er@NaYF_4_) at Site1 is calibrated under 980 nm (Figure 6c), and then, the second microchannel is filled with another UCNPs (NaYF_4_:Yb,Er@NaYF_4_:Nd,Yb) at Site2, which is calibrated under the excitations of 808 nm laser (Figure 6d) and 980 nm laser (Figure 6e) prior to in vitro and in vivo testing. Thus, the spectra of Site2 excited by a 980 nm laser under different temperature conditions can be demodulated by subtracting the spectra shown in Figure 6c from Figure 6e. The linear plots of ln(I_525_/I_545_) versus the inverse of the absolute temperature (1/*T*) in the temperature range of 30–70°C at Site1 and Site2 are shown in Figure 6f.

In order to verify that the UCNP‐FMS based on NaYF_4_:Yb,Er@NaYF_4_ and NaYF_4_:Yb,Er@NaYF_4_:Nd,Yb can realize distributed temperature detection, we immerse one detection site in hot water (60 °C) and leave the other in air, and simultaneously detect these two sites with a thermocouple (Figure 6g). The results of multi‐site temperature detection are consistent with those of traditional thermocouple measurement (Figure 6h–j). Next, we test its in vivo detection capability. Considering the brittleness of the double‐microchannel probe, we adopt an optical fiber (MMF, core/cladding 400/420 µm, NA 0.37), avoiding probe cracking during the implantations. As a proof of concept, we implant the UCNP‐FMS based on NaYF_4_:Yb,Er@NaYF_4_ and NaYF_4_:Yb,Er@NaYF_4_:Nd,Yb into the mouse brains (Figure 6k; Figure S9, Supporting Information) and detect the thermal dynamics at dual positions. When mice take food, the hippocampus and LH of mouse brains show sustained increases in temperature within 15 min (Figure 6l,m).

To further investigate the long‐term biocompatibility of the UCNP‐FMS, we test the immune response after 4 weeks of the UCNP‐FMS implantation, and find no significant inflammation and immune responses (Figure S10, Supporting Information). It suggests that the UCNP‐FMS befits as long‐term implants for temperature monitoring in vivo.

## 3 Conclusion

In summary, we propose a fiber microchannel sensor doped with thermal‐sensitive nanomaterials for real‐time temperature monitoring in vivo. For nanothermometers, the highest spatial resolution is determined by its size.^[^ ^10^ ^]^ FMS loading the nanothermometers is fabricated by femtosecond laser micromachining, ensuring high spatial resolutions. The UCNPs of high thermal sensitivity are microinjected into microchannels, whose ratiometric emission spectra are utilized for robust temperature sensing. We demonstrate that the UCNP‐FMS can detect temperature in the range of 35–80 °C with high linearity and high sensitivity (detection limit of ±0.3 °C at 37 °C). The rapid response time (∼10 s) of UCNP‐FMS makes it suitable for in vivo applications. We verify the capability of UCNP‐FMS in real‐time temperature monitoring in different brain regions of freely moving animals, under various conditions. Different from temperature imaging techniques, UCNP‐FMS can distinguish temperatures at deep brain regions.

It should also be noted that FMS outperforms conventional sol–gel techniques,^[^ ^23^ ^]^ in which sensing materials are glued on fiber tips. With FMS, it is easy to fabricate probes with no worry about environmental pollution, and it allows multi‐site distributed monitoring at high spatial resolutions. In addition to UCNPs doped with Er^3+^ ions, other rare earth ions, e.g. Tm^3+^, also have thermally coupled levels for temperature sensing, such as NaYF_4_:Yb,Tm@NaYF_4_ UCNPs (Figure S11, Supporting Information). Considering that the emission spectra of Er^3+^ doped UCNPs and Tm^3+^ doped UCNPs introduce no crosstalk to each other, these two UCNPs can be detected simultaneously by the same NIR excitation (Figure S12, Supporting Information). By combining time division multiplexing and wavelength division multiplexing technologies, temperature detection at more sites with a single probe can be realized, further expanding the application of multi‐site temperature detection in the brains. Moreover, the concept of FMS provides a broad platform for sensing various physical and chemical parameters at high spatial resolutions in vivo, which will benefit various biomedical studies.

## 4 Experimental Section

### Optical Setup for Femtosecond Laser Fabrication

The femtosecond laser fabrication device mainly included the femtosecond laser system, the optical path system, the 3D mechanical mobile platform, the real‐time monitoring system, and the computer, etc. The optical path system was mainly used to control the light beam, including processing optical path and imaging optical path. In the processing optical path, a femtosecond laser with a central wavelength of 800 nm, a pulse width of 120 fs, and a repetition frequency of 1 kHz was used in the experiment. The average power output of the laser amplifier was 4 W. The laser beam was emitted from the laser through the shutter, neutral attenuator, diaphragm, etc., and finally through the objective focused on the surface of the material to be processed. The neutral attenuator was used to adjust the energy of the laser beam, and the objective was used to focus the laser beam, so as to realize the processing of materials. The diaphragm was used to limit the laser input of the objective, so as to change the shape of the light spot and realize the control of the Gaussian beam waist radius and Rayleigh length of the focused light spot. The imaging optical path mainly consisted of a 473 nm light emitting diode (LED), tube lens, and charge‐coupled device (CCD) cameras. The surface topography of the processed fiber could be observed on the screen through the imaging system, and the processing process could be observed in real‐time. The 3D mechanical mobile platform (Newport) was used to control the moving distance and speed of the materials to be processed, with the minimum direction resolution of 0.005 µm.

### Optical Setup for Single Point Temperature Detection

A fiber‐coupled laser at 980 nm (50 mW) was used to excite the UCNPs in the microchannel of UCNP‐FMS. Silica multimode fiber (MMF, core/cladding 200/220 µm, NA 0.37, Inper) was connected to the UCNP‐FMS through ceramic sleeves to achieve laser excitation and emission collection through a 50:50 fiber coupler. The collected emissions of the UCNP‐FMS were measured by a spectrometer (Thorlabs, CCS100, the detection range was 350–700 nm). A short‐pass optical filter with a cutoff wavelength of 850 nm was employed to suppress the incidence of the residual excitation laser into the spectrometer. To characterize its temperature response, the UCNP‐FMS was stored in a digital incubator equipped with a thermocouple (resolution, 0.1 °C). In order to reduce the influence of thermal effect on in vivo temperature detection, optical fiber mechanical switch (1 Hz, the duty cycle is 0.5) was adopted.

### Optical Setup for Two‐Point Temperature Detection

The two fiber‐coupled lasers at 980 nm (50 mW) and at 808 nm (50 mW) were coupled into a 50:50 fiber coupler, used for exciting the two kinds of UCNPs in the microchannels of UCNP‐FMS. The two lasers were modulated by time division multiplexing through LabVIEW program and data acquisition card. Specifically, the 980 and 808 nm lasers were modulated by square wave of 0.1 Hz with the 20% duty ratio, and the phase difference was π. Silica multimode fiber (MMF, core/cladding 400/420 µm, NA 0.37, Inper) was connected to the UCNP‐FMS through ceramic sleeves to achieve laser excitation and emission collection through a 50:50 fiber coupler. The collected emissions of the UCNP‐FMS were measured by a spectrometer (Thorlabs, CCS100, the detection range was 350–700 nm). A short‐pass optical filter with cutoff wavelength of 600 nm was employed to suppress the incidence of the residual excitation laser into the spectrometer. To characterize its temperature response, the UCNP‐FMS was stored in a digital incubator equipped with a thermocouple (resolution, 0.1 °C).

### Animal Husbandries

C57/BL6 wild male mice (WT, 8–9 weeks old) were obtained from Charles River Laboratories (Beijing, China). They were maintained in a temperature‐controlled room on a 12‐h light/dark cycle (lights on 06:00–18:00) with ad libitum access to food and water and fed a standard diet. All procedures for animal surgery and experimentation were performed using protocols approved by the Institutional Animal Care and Use Committee (IACUC) at the Tsinghua University.

### In Vivo Implantation of UCNP‐FMS

The surgical tools were sterilized before surgery. Mice were under anesthetization with isoflurane: 3.5% v v^−1^ for induction and 1.5% v v^−1^ for maintenance, heating pads were used to maintain the mouse body temperature. Mouse eyes were covered with ophthalmic ointment to prevent drying and the mice were mounted in a stereotaxic frame with ear bars. A surgical area with electric shave was cleared. A scalpel blade was used to cut and remove the skin over the skull surface and remove the periosteum to expose and clean the surface of the skull. Used an empty glass pipet to bregma and record the antero‐posterior (A/P) and medio‐lateral (M/L) coordinated to complete the calibration process. The sites as follows: mPFC of cortex A/P +0.97 mm, M/L ±1 mm, D/V −0.66 mm, hippocampal CA1 A/P −2.15 mm, M/L ±1.55 mm, D/V −1.25 mm and LH A/P −1.67 mm, M/L ±0.9 mm, D/V −4.5 mm. For dual‐microchannel probes, the sites as follows: hippocampus A/P −1.67 mm, M/L ±0.9 mm, D/V ‐1 mm, and LH A/P −1.67 mm, M/L ±0.9 mm, D/V −4.5 mm. Aperture on the skull using a miniature hand‐held skull drill (68 605, RWD Life Science) and used PBS buffer to clean blood stains. The UCNP‐FMS was moved to targeted areas and completed the implantation. After the site was sealed with dental cements mixed with toners. The rest of the skull surface was covered as well, making sure the edges of the skin were covered by cement and letting it dry. Last, isoflurane administration was stopped and the mouse was left in a cage until it fully recovers. The mouse was injected with 5% w v^−1^ glucose in saline for rehydration and 0.1 mg kg^−1^ buprenorphine (*i.p*., instant release) for post‐operative analgesia. To minimize potential immunological reaction, the mice were injected daily with 20 µl per 100 g cyclosporine (*i.p*.) since the day before implantation.

### In Vivo Temperature Monitoring

All test indexes were performed after the complete recovery of mice. Food intake: The day before the formal experiment, mice were required to fast for 16 h (5:00 pm–9:00 am) with water available before feeding experiments.^[^ ^37^ ^]^ The implanted FOMS doped with UCNPs were connected to the sensing system to detect the baseline of freely moving mice. Then mice were given food freely and the dynamic changes in brain temperature in LH were recorded. Anesthesia/wakefulness: At the beginning, the baseline brain temperature of the freely moving mice was recorded for 5 min. And then isoflurane was used to induce anesthesia for 10 min. Finally, isoflurane administration was stopped, and waited the mice for gradually recovering. The brain temperature in CA1 was recorded during the test. External temperature changes: To determine the dynamic changes of brain temperature caused by environmental changes, the mice was first placed in the home‐cage covered with a heating pad (5 min), and continuously monitored the dynamic change process. Then, the heating pad was removed, and gently treated the mice's body except for the head with atomized ethanol (5 min). The heat treatment experiment (5 min) was repeated to test the brain temperature changes in mPFC of cortex.

### Morphological Identification and Immunofluorescence Examination

Animals were deeply anesthetized, then transcardially perfused with 0.1 m phosphate‐buffered saline (PBS), followed by 4% paraformaldehyde‐borate fixative (PFA, pH 7.4). Tissues were removed and post‐fixed in the PFA 12–14 h at 4 °C, cryoprotection in 30% sucrose solution before for section making. Tissues were sliced into 30 µm sections with a cryostat microtome (CM3050 S, Leica). Sections were washed by PBS and initial blocking (3% normal donkey serum in 0.01 m PBS containing 1% Triton X‐100), and incubated with the primary antibodies overnight at 4 °C, i.e., Rabbit anti‐NeuN (1:1000; ab177487, Abcam) and Mouse anti‐Sox9 (1:1000; ab76997, Abcam). Then the brain sections were washed and incubated with Alexa Fluor 647 Donkey anti‐Mouse IgG (1:1000, ab150107, Abcam) and Alexa Fluor 555 Donkey anti‐Rabbit IgG (1:1000, ab150062, Abcam) for 1.5 h at room temperature. Last, the brain sections were cover slipped with VECTASHIELD mounting media containing DAPI (F6057, Sigma) and images were captured by a confocal microscope (FV3000, Olympus).

### Statistical Analysis

All the statistical tests were two‐tailed and performed in MATLAB (R2012b). Except where indicated otherwise, all summary data were presented as the mean ± SEM. Group differences were analyzed using the paired or unpaired student's t‐test (GraphPad Prism 7 San Diego, CA, USA). Results with *P* values of <0.05 were considered statistically significant.

## Conflict of Interest

The authors declare no conflicts of interest.

## Author Contributions

B.Z. and K.F. contributed equally to this work. L.K. conceived the idea and supervised the project. B.Z., K.F., J.Z., and C.J. performed the experiments. B.Z., K.F., and L.K. analyzed the data. All authors contributed to the editing of the manuscript.

## Supporting information

Supporting Information

## Data Availability

The data that support the findings of this study are available from the corresponding author upon reasonable request.
