# Supplementary material for: Upconversion‐Luminescent Fiber Microchannel Sensors for Temperature Monitoring at High Spatial Resolution in the Brains of Freely Moving Animals
Source: Adv Sci (Weinh). 2023 Sep 15;10(30):2303527. doi: 10.1002/advs.202303527 (PMC10602553; doi:10.1002/advs.202303527)
Supplement: Supplementary file 1 — Supporting Information [file ADVS-10-2303527-s001.pdf]

ADVANCED SCIENCE
Open Access

Supporting Information

for *Adv. Sci.*, DOI 10.1002/advs.202303527

Upconversion-Luminescent Fiber Microchannel Sensors for Temperature Monitoring at High Spatial Resolution in the Brains of Freely Moving Animals

*Bingqian Zhou, Kuikui Fan, Jiazhen Zhai, Cheng Jin and Lingjie Kong**

Supporting Information

Upconversion-luminescent fiber microchannel sensors for temperature monitoring at high spatial resolution in the brains of freely moving animals

*Bingqian Zhou, Kuikui Fan, Jiazhen Zhai, Cheng Jin, and Lingjie Kong**

E-mail: konglj@tsinghua.edu.cn (L.K.)

Optimized processing of femtosecond laser micromachining

Femtosecond laser processing parameters are optimized by controlling the laser power and the diaphragm diameter. We focus the laser on the surface of the MMF (core/cladding: 200/220 μm) and begin the laser processing within 1s by opening the shutter. In the laser processing, the diaphragm is used for spot shaping, improving the integrity of the processed structure and reducing the material cracks caused by processing. The diameters of diaphragm are set at 4 mm, 6 mm, 8 mm, and 10 mm, making the light spot before objective 1 fill or underfill the back pupil (Diameter: 10 mm) of the objective 1 in Figure 2a. As shown in Figures S1a, b (Supporting Information), the diameters of diaphragm affect the microchannel aperture and depth, because the decrease of the diaphragm diameter reduces the actual NA of objective 1, thus leading to the increase of the aperture and depth of the microchannel under the same laser power. With the increase of the laser power, the microchannel aperture and depth increase gradually and saturate at 150 mW. Under the laser power of 60 mW, the microchannel aperture is about 30 μm, and the microchannel depth is about 100 μm. With the increase of processing time and the movement of the MMF along *z* axis in Figure 2a, the microchannel aperture and depth increase. We choose 50 μm microchannel diameter, which is enough for doping UCNPs and meets the hardness of implants. Therefore, we adopt the diameter of diaphragm as 4 mm and the laser power as 60 mW.

Characterization of temperature-sensitive UCNPs based on $NaYF_4$:Yb,Er@$NaYF_4$:Nd,Yb

Figure S5a (Supporting Information) shows the schematic diagram of the upconversion process of the $NaYF_4$:Yb,Er@$NaYF_4$:Nd,Yb nanoparticles. The $NaYF_4$:Yb,Er@$NaYF_4$:Nd,Yb UCNPs generates 525 nm and 545 nm emissions under 808 nm laser excitation via multiple energy transfers from Nd^{3+} ions to Yb^{3+} ions, and Yb^{3+} ions to Er^{3+} ions. The TEM image of the $NaYF_4$:Yb,Er@$NaYF_4$:Nd,Yb UCNPs is shown in Figure S5b (Supporting Information) with the diameter of about 60 nm. We demonstrate the performance of UCNP-FMS based on $NaYF_4$:Yb,Er@$NaYF_4$:Nd,Yb in temperature sensing *in vitro*, as shown in Figure S5c (Supporting Information), and find that the emission spectra are highly dependent on the temperature, attributed to the thermally coupled energy states of Er^{3+}, which is similar to $NaYF_4$:Yb,Er@$NaYF_4$ UCNPs. A linear plot of $\ln(I_{525}/I_{545})$ versus the inverse of the absolute temperature ($1/T$) in the temperature range of 25 °C to 80 °C is shown in Figure S5d (Supporting Information).

Spectral demodulation method in distributed temperature sensing process

Figure S8 (Supporting Information) shows the schematic diagram of the spectral demodulation during distributed temperature sensing process. Firstly, the temperature calibration of the two-microchannel fiber probe should be carried out. UCNPs 1 ($NaYF_4$:Yb,Er@$NaYF_4$) is added at Site1, which can be excited by 980 nm laser but cannot be excited by 808 nm laser. Temperature calibration spectrum is achieved at Site1 under 980 nm laser excitation (Figure 6c). At Site2, UCNPs 2 ($NaYF_4$:Yb,Er@$NaYF_4$:Nd,Yb) is added, which can be excited at both 980 nm and 808 nm lasers. When excited by 808 nm laser, the calibrated spectrum corresponding to the temperature at Site2 is achieved (Figure 6d). Then, 980 nm laser is used for exciting the two positions, resulting in a superposition of the spectra of the two positions (Figure 6e). In the actual detection process, we excite the probe by 808 nm and 980 nm lasers separately, and get two spectra (Spectrum2@Site2 and Spectrum3@Site1&Site2). According to the 808 nm excitation spectrum (Spectrum2@Site2), the temperature of Site2 can be determined. Then, according to the previous calibration data (Figures 6c, e), the spectrum of Site2 under the 980 nm excitation can be calculated. Then, the spectral information at Site1 can be obtained by subtracting the spectra at Site2 from the spectra obtained by the excitation at 980 nm (Spectrum3@Site1&Site2), and the temperature at Site1 can be calculated accordingly.

Characterization of temperature-sensitive UCNPs based on $NaYF_4$:Yb,Tm@$NaYF_4$

Figure S11a (Supporting Information) shows the schematic diagram of the upconversion process of the $NaYF_4$:Yb,Tm@$NaYF_4$ nanoparticles. The core of $NaYF_4$:Yb,Tm@$NaYF_4$ UCNPs provides 700-800 nm emissions under 980 nm excitations via energy transfer from the Yb^{3+} ions to Tm^{3+} ions. The TEM image of the $NaYF_4$:Yb,Tm@$NaYF_4$ UCNPs is shown in Figure S11b (Supporting Information) with the diameter of about 30 nm. We demonstrate the performance of UCNP-FMS based on $NaYF_4$:Yb,Tm@$NaYF_4$ in temperature sensing *in vitro*, as shown in Figure S11c (Supporting Information), and find that the emission spectra are highly dependent on the temperature, attributed to the thermally coupled energy states of Tm^{3+}. The $^3F_2$, $^3F_3$→$^3H_6$ and $^3H_4$→$^3H_6$ transitions also comply with the thermal equilibrium governed by the Boltzmann factor, described as

$$I_{700}/I_{800} = C\exp(-\Delta E/kT) \qquad (1)$$

where I_{700} and I_{800} are the emission intensities of the $^3F_2$, $^3F_3$→$^3H_6$ and $^3H_4$→$^3H_6$ transitions, respectively; C is a constant determined by the host material of UCNPs; ΔE is the energy gap between the $^3F_2$, $^3F_3$ and the $^3H_4$ states; k is the Boltzmann constant; T is the absolute temperature (in Kelvin scale). A linear plot of $\ln(I_{700}/I_{800})$ versus the inverse of the absolute temperature ($1/T$) in the temperature range of 30 °C to 90 °C is shown in Figure S11d (Supporting Information).

Distributed temperature sensing with Er^{3+} and Tm^{3+} doped UCNP-FMS

We dope two kinds of UCNPs, *i.e.* $NaYF_4$:Yb,Er@$NaYF_4$ UCNPs and $NaYF_4$:Yb,Tm@$NaYF_4$ UCNPs, into two microchannels along a FMS probe (Figure S12a, Supporting Information), considering that the emission spectra of two UCNPs introduce no crosstalk to each other. This makes it possible to simultaneously monitor temperatures at distributed positions with a single FMS probe. We test the *in vitro* temperature response of the UCNP-FMS based on $NaYF_4$:Yb,Er@$NaYF_4$ and $NaYF_4$:Yb,Tm@$NaYF_4$ (Figure S12b, Supporting Information) and calibrate the linear responses of both UCNPs range from 30 ℃ to 70 ℃ (Figure S12c, Supporting Information).

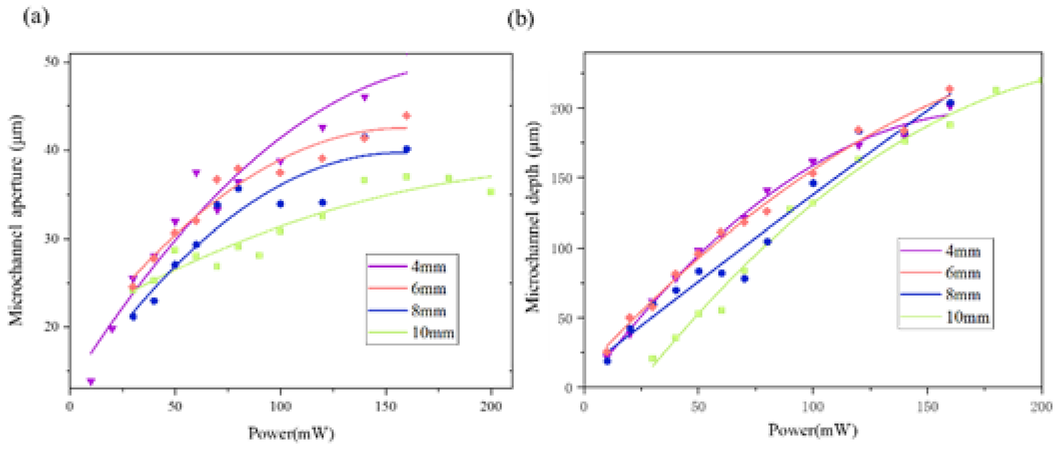

Figure S1. Optimized processing of femtosecond laser micromachining. a) Effects of laser power on the microchannel aperture for different diaphragm diameters (4 mm, 6 mm, 8 mm, and 10 mm). b) Effect of laser power on microchannel depth for different diaphragm diameters (4 mm, 6 mm, 8 mm, and 10 mm).

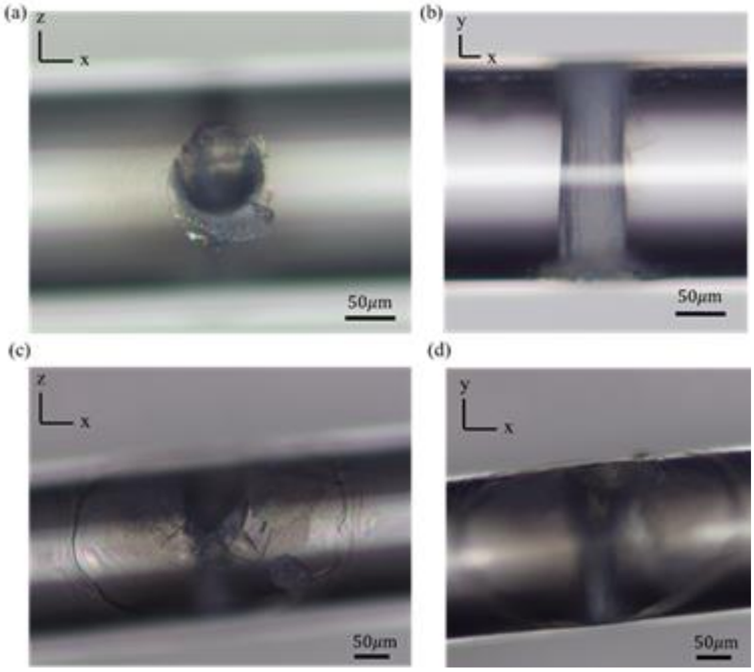

Figure S2. Photos of microchannels on the FMS, without (a, b) and with (c, d) injecting UCNPs. (a) and (c): *x*-*z* plane view; (b) and (d): *x*-*y* plane view.

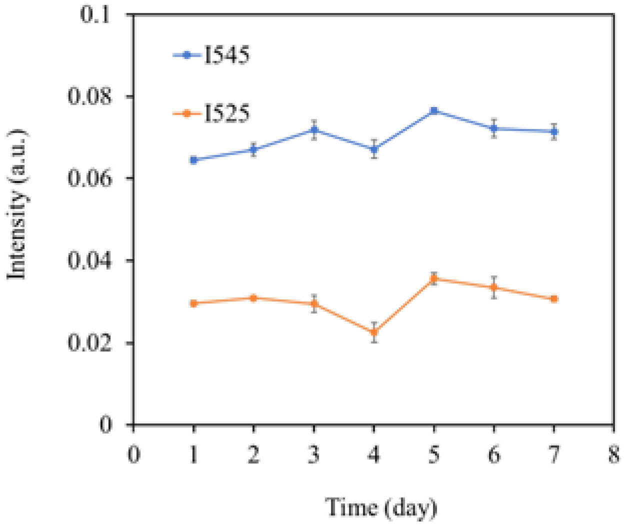

Figure S3. Long-term stability of the UCNP-FMS in mouse brains *in vivo*. The sensors are implanted in cerebral cortexes of mouse brains. Intensities of two emission bands are recorded for a week (n=3). Data are presented as mean ± SEM, n = 3 per group.

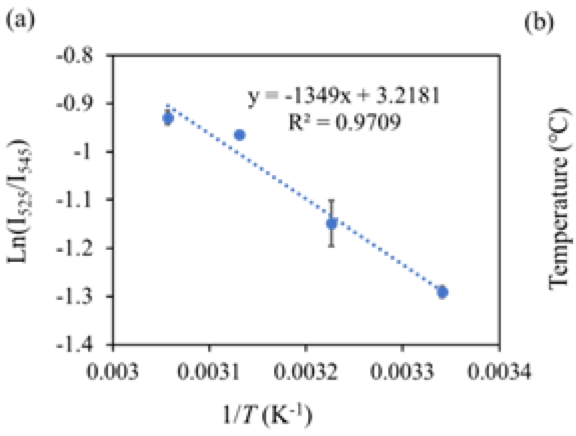

Figure S4. Temperature calibration of the UCNP-FMS. (a) The temperature calibration of the UCNP-FMS *in vitro* by thermocouple (YET-620, YOWEXA). (b) The temperature calibration of the UCNP-FMS in mouse brains *in vivo* by thermocouple (n=3). For statistical results, values with error bars indicate mean ± SEM, n = 3 mice per group. P values: n.s. denotes $p>0.05$ (student’s t test).

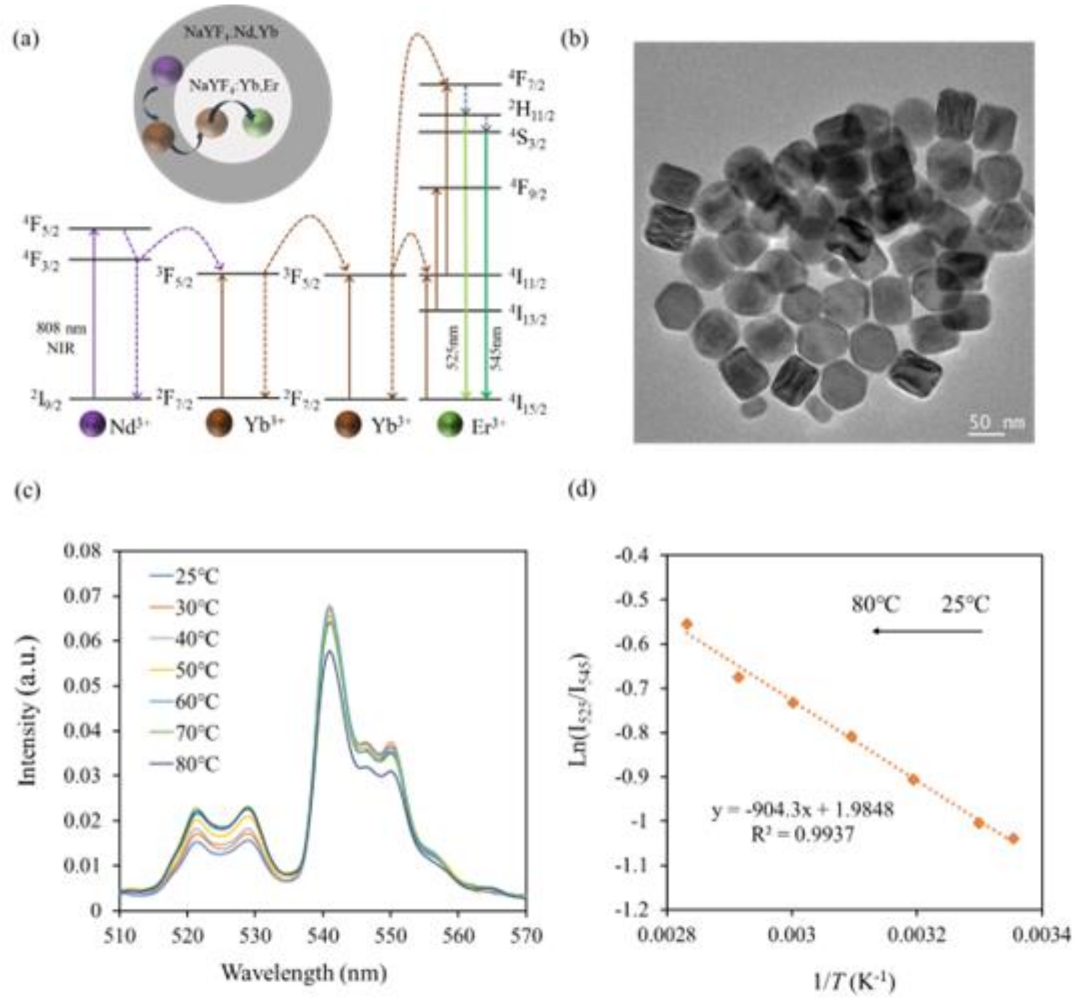

Figure S5. Characterization of UCNP-FMS based on $NaYF_4$:Yb,Er@$NaYF_4$:Nd,Yb. a) Schematics of the upconversion process of $NaYF_4$:Yb, Er@$NaYF_4$:Nd,Yb UCNPs. The energy level diagram shows the upconversion process. b) TEM image of the $NaYF_4$:Yb,Er@$NaYF_4$:Nd,Yb UCNPs. Scar bar: 50 nm. c) Emission spectra of FMS doped with $NaYF_4$:Yb,Er@$NaYF_4$:Nd,Yb UCNPs at various temperatures. d) Linear relationships between ln(I_{525}/I_{545}) and the inverse absolute temperature (1/T) in the range of 25-80 °C. The wavelength of excitation laser is 808 nm with 15 mW.

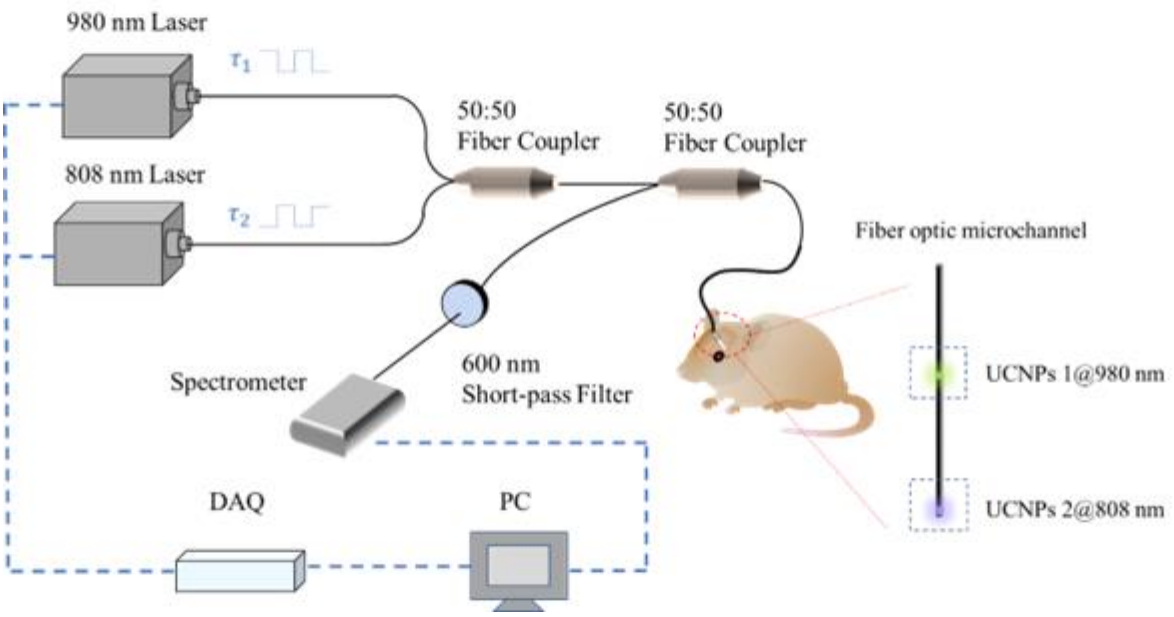

Figure S6. Optical setup for dual-site temperature sensing of UCNP-FMS based on $NaYF_4$:Yb,Er@$NaYF_4$ and $NaYF_4$:Yb,Er@$NaYF_4$:Nd,Yb. DAQ, data acquisition card; PC, personal computer. UCNPs 1@980 nm denotes $NaYF_4$:Yb,Er@$NaYF_4$ UCNPs excited by 980 nm laser. UCNPs 2@808 nm denotes $NaYF_4$:Yb,Er@$NaYF_4$:Nd,Yb UCNPs excited by 808 nm laser. Through time division multiplexing, dual-site temperature detection is achieved. τ_1 and τ_2 denote the time modulations of 980 nm laser and 808 nm laser.

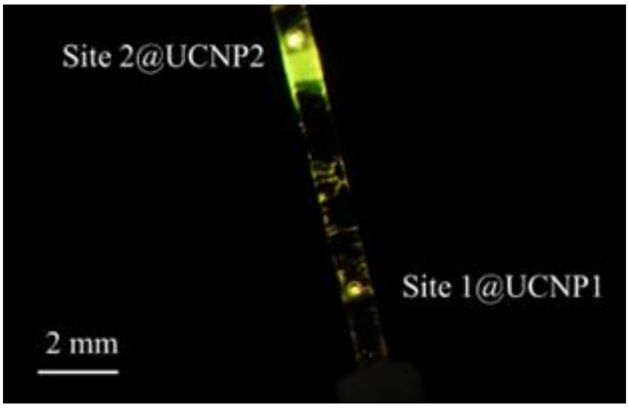

Figure S7. UCNP-FMS doped with two UCNPs excited by 980 nm laser (15 mW). UCNPs 1: $NaYF_4$:Yb,Er@$NaYF_4$, UCNPs 2: $NaYF_4$:Yb,Er@$NaYF_4$:Nd,Yb.

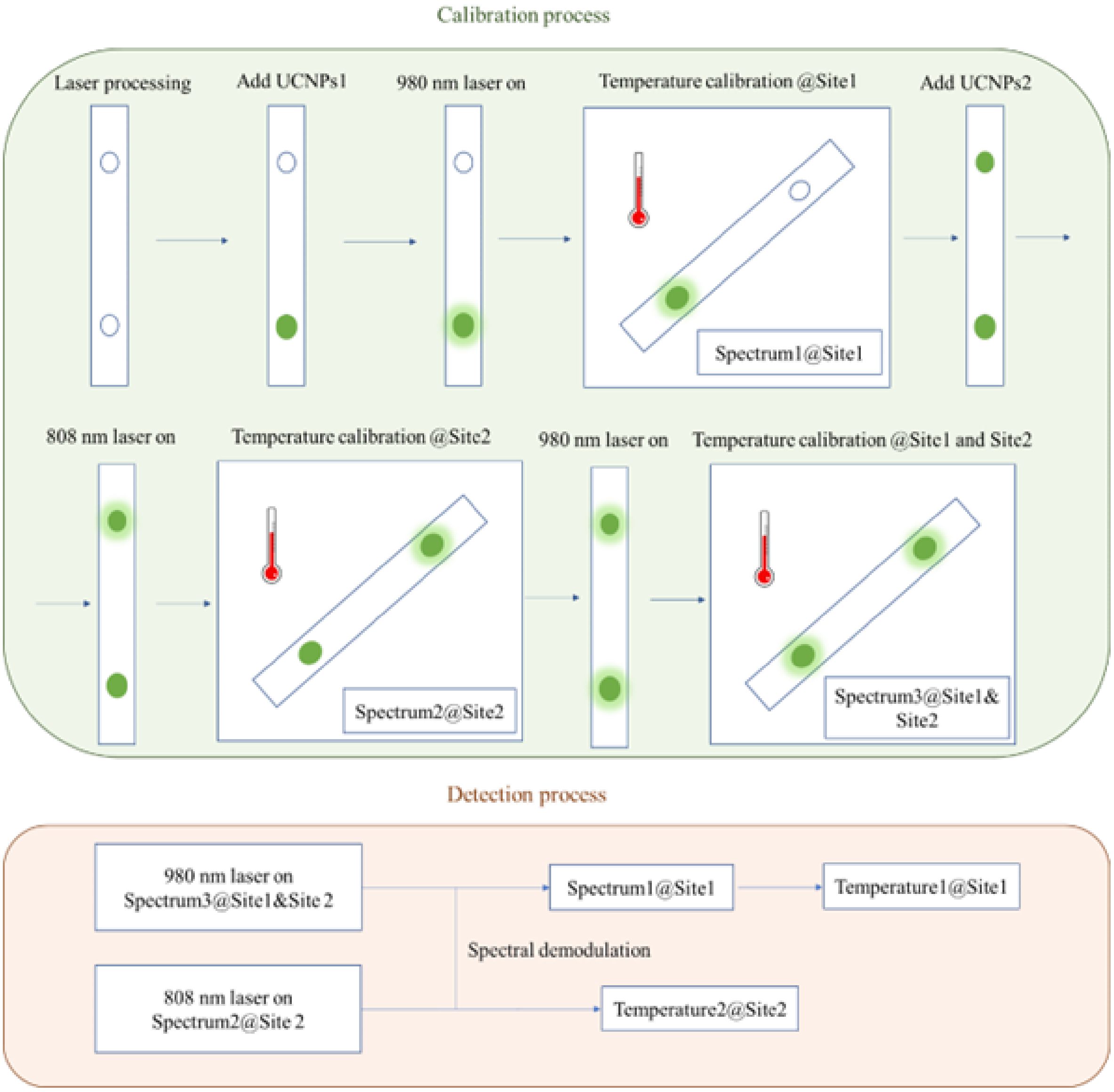

Figure S8. Spectral demodulation method in distributed temperature sensing process. UCNPs1: $NaYF_4$:Yb,Er@$NaYF_4$, and UCNPs2: $NaYF_4$:Yb,Er@$NaYF_4$:Nd,Yb.

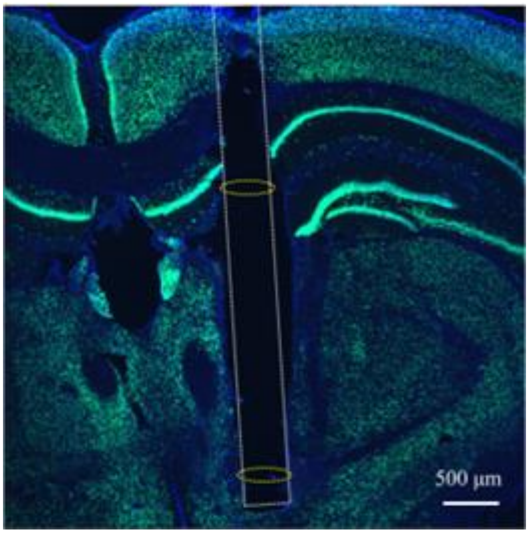

Figure S9. Localization map of UCNP-FMS based on $NaYF_4$:Yb,Er@$NaYF_4$ and $NaYF_4$:Yb,Er@$NaYF_4$:Nd,Yb implantation. The dotted box in white is the location of UCNP-FMS. The dotted boxes in yellow are the microchannnels of the probe loacated at hippocampus and LH. Blue: DAPI, green: NeuN.

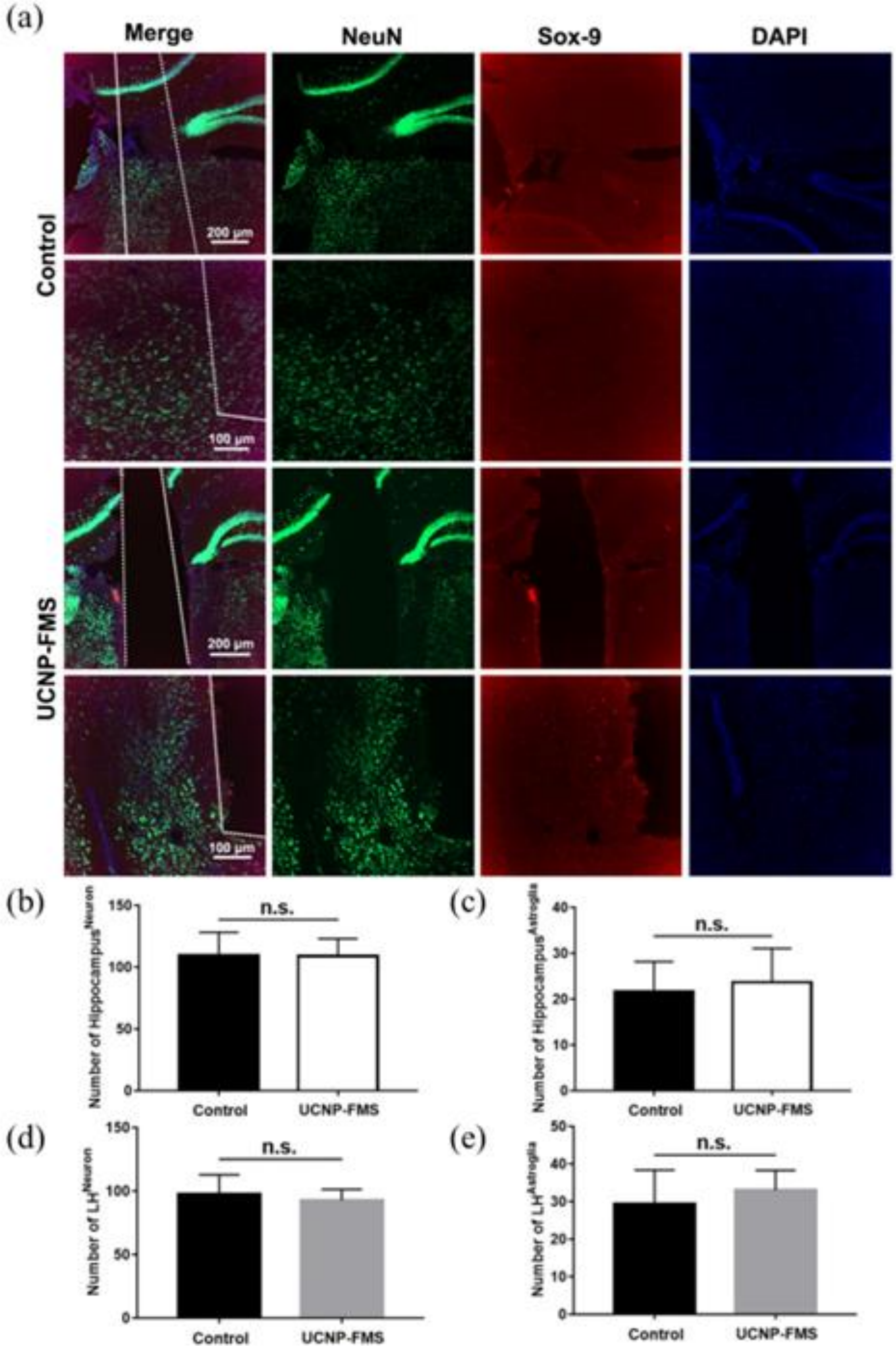

Figure S10. Immunoassay of the mouse brain perfused at Day 28 after the implantation of UCNP-FMS based on $NaYF_4$:Yb,Er@$NaYF_4$ and $NaYF_4$:Yb,Er@$NaYF_4$:Nd,Yb. a) Immunostaining of the control group (the contralateral brain regions) and the implanted group. The first and third rows correspond to the position of the first microchannel of the probe. The second and fourth rows correspond to the position of the second microchannel of the probe. Red: SOX9 labelling astroglia; green: NeuN labelling neurons; blue: DAPI labelling nuclei.

The dotted box indicates the implanted location of the UCNP-FMS. b, c) Quantitative comparisons of the number of astroglia and survived neurons around the first microchannel (near the hippocampus) of the probe referred by SOX9 (c) and NeuN (b) staining, respectively (n = 3). d, e) Quantitative comparisons of the number of astroglia and survived neurons around the second microchannel (near the LH) of the probe referred by SOX9 (e) and NeuN (d) staining, respectively (n = 3). P values: n.s. denotes $p>0.05$ (student's t test). Data are presented as mean ± SEM, n = 3 per group.

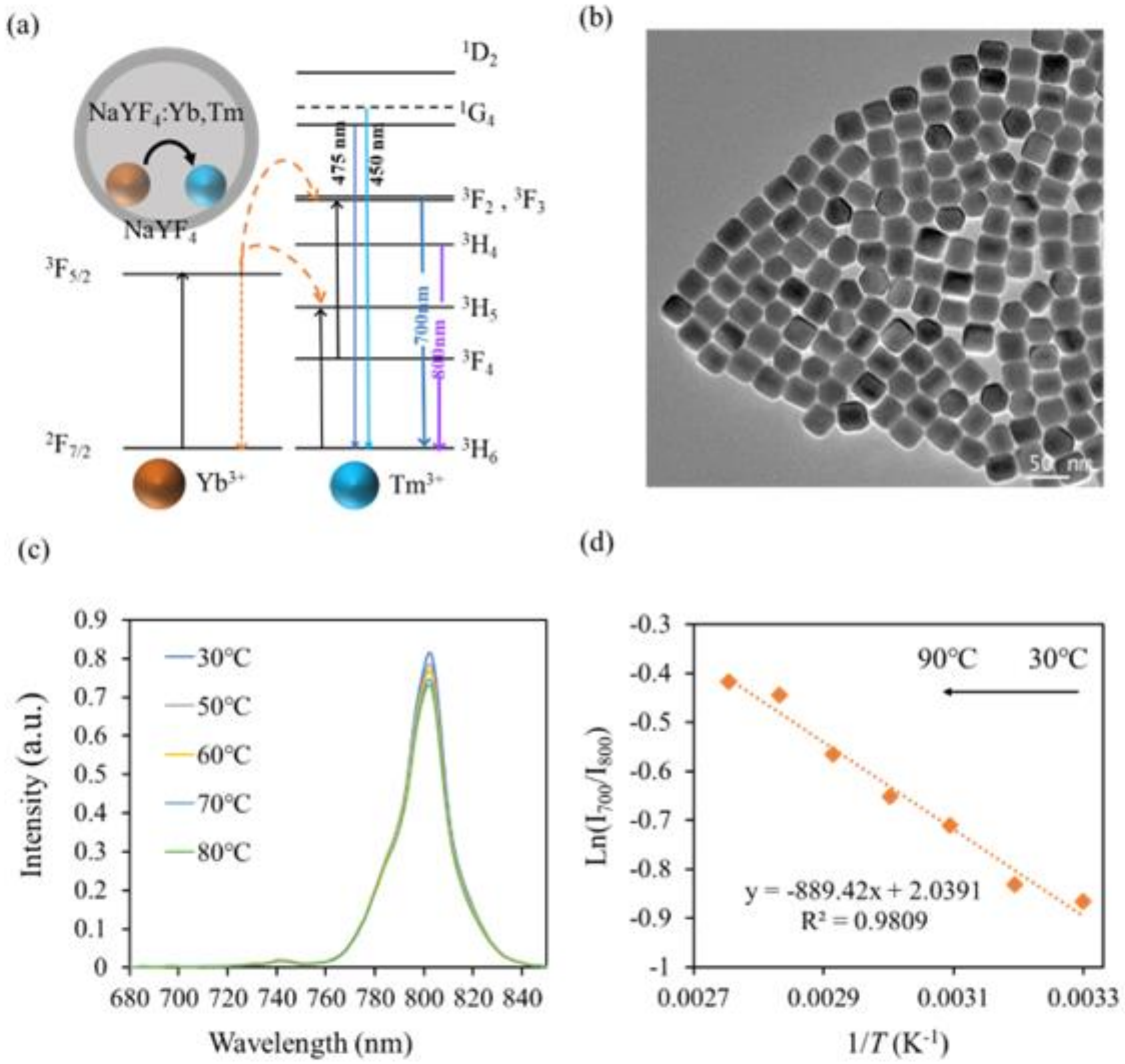

Figure S11. Characterization of UCNP-FMS based on $NaYF_4$:Yb,Tm@$NaYF_4$. a) Schematics of the upconversion process of $NaYF_4$:Yb,Tm@$NaYF_4$ UCNPs. The energy level diagram shows the upconversion process. b) TEM image of the $NaYF_4$:Yb, Tm@$NaYF_4$ nanoparticles. Scar bar: 50 nm. c) Emission spectra of FMS doped with $NaYF_4$:Yb,Tm@$NaYF_4$ UCNPs at various temperatures. d) Linear relationships between $\ln(I_{700}/I_{800})$ and the inverse absolute temperature (1/*T*) in the range of 30-90 °C. The wavelength of excitation laser is 980 nm with 20 mW.

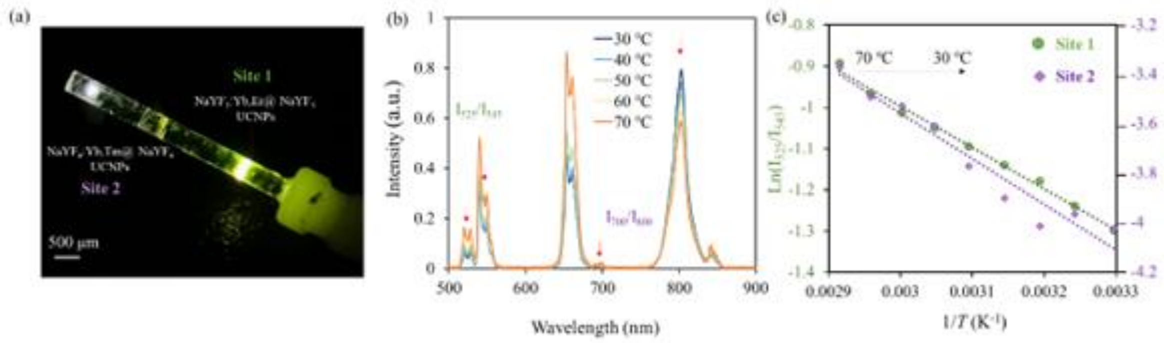

Figure S12. Distributed temperature sensing *in vitro* with UCNP-FMS based on $NaYF_4$:Yb,Er@$NaYF_4$ and $NaYF_4$:Yb,Tm@$NaYF_4$. a) FMS probe doped with $NaYF_4$:Yb, Er@$NaYF_4$ nanoparticles and $NaYF_4$:Yb, Tm@$NaYF_4$ nanoparticles at seperate microchannels. b) Emission spectra of FMS doped with $NaYF_4$:Yb,Er@$NaYF_4$ and $NaYF_4$:Yb,Tm@$NaYF_4$ UCNPs at various temperatures. c) Linear relationships of two kinds of UCNPs between ln(I_{525}/I_{545}) or ln(I_{700}/I_{800}) and the inverse absolute temperature (1/*T*) in the range of 30-70 °C. The wavelength of excitation laser is 980 nm with 20 mW.
